# Evaluation of literature searching and article selection skills of an evidence-based practice team

**DOI:** 10.5195/jmla.2020.865

**Published:** 2020-07-01

**Authors:** Emily Paige Jones, Emily A. Brennan, Amanda Davis

**Affiliations:** 1 jonesemi@musc.edu, Research and Education Informationist, MUSC Libraries, and Instructor, Academic Affairs Faculty Department, Medical University of South Carolina, Charleston, SC; 2 brennane@musc.edu, Research and Education Informationist, MUSC Libraries, and Associate Professor, Academic Affairs Faculty Department, MUSC Libraries, Medical University of South Carolina, Charleston, SC; 3 davisam@musc.edu, Senior Evidence-Based Practice Analyst, Value Institute, MUSC Health, Medical University of South Carolina, Charleston, SC

## Abstract

**Background:**

An evidence-based practice (EBP) team at an academic medical center supports the development of evidence-based hospital policies and protocols via “Evidence Briefs.” An early career librarian was added to the EBP team to meet increased requests for Evidence Briefs, which provided an opportunity to initiate a quality improvement (QI) analysis, improve work flow, and cross-train staff on literature searching and article selection skills.

**Case Presentation:**

This QI project evaluated literature searching and article selection skills of an early career librarian (less than 2 years' experience), a mid-career librarian (more than 10 years' experience), and a critical appraisal expert. This project examined 10 Evidence Brief requests completed within a 6-month period. Analysis of each individual's performance of literature searching and article selection was completed for each Evidence Brief. Across all Evidence Brief requests, the mid-career librarian performed the most comprehensive literature searches and captured the highest number of articles that ultimately ended up being included in Evidence Briefs (75%). The critical appraisal expert performed best on the article selection portion of the project and identified the highest number of relevant articles that were included in Evidence Briefs (74%).

**Conclusions:**

This project provided a formalized method of assessing the literature searching and article selection skills of each member of the EBP team. This project illustrated the skill level of each individual and led to improvements in the Evidence Brief request work flow.

## BACKGROUND

At the authors' academic health system, the Value Institute collaborates with care teams by providing data, evidence, and tools to improve health care value. The Value Institute, which resides in the hospital's Quality Department, provides quality analytics and evidence-based practice (EBP) support to create a culture of data-driven and evidence-based decision making. One way the Value Institute supports clinical inquiry and integration of EBP at the bedside is through developing clinical decision support (CDS) tools. CDS tools aim to improve quality of care through the Five Rights model: communicating the right information to the right stakeholder, at the right point in the work flow, through the right channel, and the right format [1]. The Value Institute creates CDS tools using both a top-down model informed by senior leaders and a grassroots model of requests from frontline staff.

The Value Institute's EBP team supports the development of evidence-based hospital policies and protocols via “Evidence Briefs.” Evidence Briefs include a narrative summary and a table of best evidence for a single clinical question to assist with clinical decision making; however, they do not directly offer practice recommendations. Evidence Briefs are developed using comprehensive literature search methods and thorough critical appraisal of best evidence in an attempt to provide an objective summary of research in a clear and useful manner [2]. A sample Evidence Brief can be found in supplemental Appendix A. The EBP team has offered an Evidence Brief service since 2014. This service began as a collaboration with clinical informatics to assist with triaging change requests and has expanded to support clinical inquiry for frontline staff.

Frontline staff must specify the following items during the Evidence Brief request process:

1. Relationship of request to clinical or administrative policy update
2. Problem or situation in need of investigation
3. Relationship of problem of interest to unit or hospital goals
4. Explanation of current process
5. Patient/population/problem, intervention, comparison, outcome (PICO) elements

One member of the EBP team, the critical appraisal expert, triages requests on a first-come, first-served basis and seeks clarification from the requestor before transferring the request to the medical librarian for literature review. Once the medical librarian completes the literature search and identifies pertinent articles for the clinical question, the search strategy and selected articles are emailed to the critical appraisal expert. Then, an Evidence Brief is created by critically appraising relevant articles, and the results are provided to the requestors for discussion with key stakeholders. While the “question and answer plus critical appraisal” model [3] is not unique to clinical librarianship, this Evidence Brief service differs in that it provides a transparent and formal appraisal of the literature using a modified Grading of Recommendations Assessment, Development and Evaluation (GRADE) criteria [4, 5].

At the inception of the Evidence Brief service, the EBP team comprised the mid-career librarian and the critical appraisal expert. The inclusion of a librarian on the EBP team ensured that search strategies were structured to retrieve as many relevant studies as possible, which would ultimately result in a lower risk of bias in the overall body of literature used to make decisions [6]. The critical appraisal expert was formally trained in the use of the GRADE criteria to evaluate literature and identify themes for an entire body of literature, rather than individual studies. She is a registered dietitian with a master of public health degree and both clinical and research experience. The critical appraisal expert gained her skills through her hands-on work as a research associate and institutional review board coordinator, as well as through educational training in research methodology. She has been a critical appraisal expert with the Value Institute for five years, where she facilitates evidence-based decision making with interprofessional health care teams using techniques aligned with those used in the Evidence Brief service. During this time, the medical librarian trained her in literature searching techniques.

The volume of Evidence Brief requests grew more than 300% from 8 requests in 2014 to 35 in 2018, due to increased collaboration with nursing Shared Governance (a Magnet hospital requirement) and word-of-mouth testimony from satisfied requestors. The process established in 2014 underwent very few modifications as the program grew, except for the development of an online request form. Increased utilization of this service has improved patient care across the clinical enterprise; however, it has also placed a strain on the bandwidth of the small EBP team. To increase the program's ability to complete additional incoming requests in a timely manner, the early career librarian was added to the team in 2018.

Prior to the addition of the early career librarian, there had been no evaluation of the literature searching and article selection components of the Evidence Brief process. The addition of a new librarian provided an opportunity to initiate a quality improvement (QI) analysis, improve work flow, and cross-train staff on literature searching and article selection skills.

## CASE PRESENTATION

### Overview of the quality improvement project

A QI project was initiated to evaluate literature searching and article selection skills of the early career librarian, who had less than two years' experience; the mid-career librarian, who had more than ten years' experience; and the critical appraisal expert. This project examined ten Evidence Brief requests that were completed within a six-month period. For every Evidence Brief request, the early career librarian, mid-career librarian, and the critical appraisal expert developed individual search strategies. Then, each person selected what they considered to be the most relevant articles to answer the clinical question. These articles were combined into one RefWorks folder and de-duplicated. The critical appraisal expert reviewed the RefWorks folder and determined—based on study design, direct applicability, and actionability—which articles to include in the Evidence Brief.

### Literature searching process

Each EBP team member developed search strategies for the ten Evidence Brief requests. The number of databases that were searched and complexity of the search strategies varied depending on the clinical question. Search strategies incorporated a combination of subject headings (e.g., Medical Subject Headings [MeSH] in PubMed) and keywords for each concept in the clinical question.

PubMed was typically searched first, and that search strategy was modified for the other databases by replacing MeSH terms with appropriate subject headings (when available) and maintaining similar keywords. Supplemental databases that were routinely searched included Scopus, CINAHL Complete, PsycINFO, and Cochrane Database of Systematic Reviews. Resources that were less commonly searched included ECRI Guidelines Trust, ProQuest Health Management Database, TRIP Database, Google Scholar, and Google, among others. Filters such as publication date, study design, and ages were applied, depending on the clinical question and extent of relevant literature. To identify additional articles, the reference lists of relevant articles were hand searched, as well as citing articles.

After the literature search for each clinical question was completed, each EBP team member recorded in a Microsoft Excel spreadsheet their respective search strategy for each database searched, as well as any filters that they applied.

**Figure 1:**
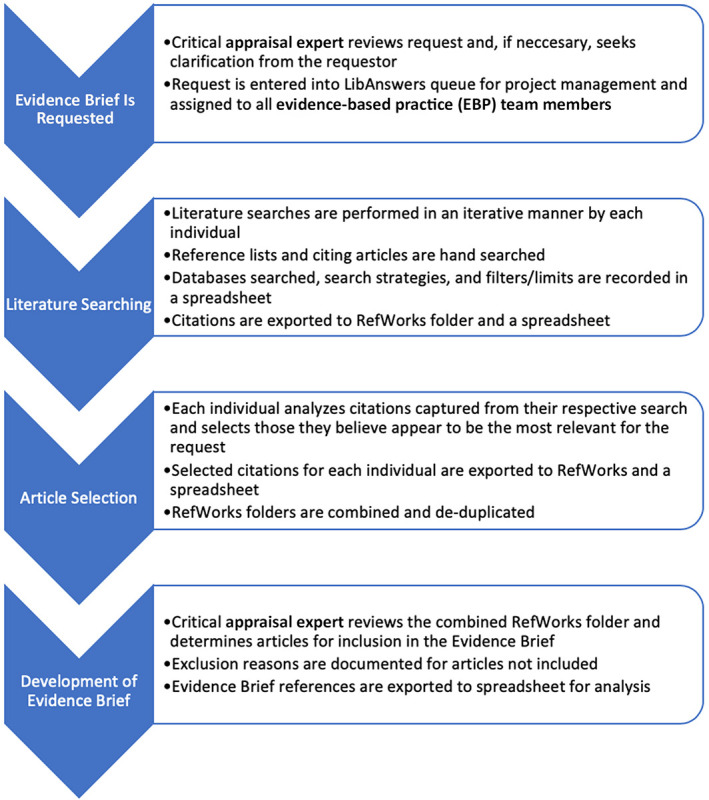
Flowchart of the process for the quality improvement (QI) project

Each person exported all retrieved citations from their final search strategies for each database and any citations discovered through hand searching into the individual's “search results” RefWorks folder and Microsoft Excel spreadsheet. A flowchart of the process for the QI project can be found in Figure 1.

### Article selection process

After completing the literature searching process, each person then selected what they considered to be the most relevant articles to answer the clinical question. Article relevance was based on a variety of considerations, such as direct applicability to the PICO elements in the clinical question, as well as study design, publication date, and actionability (e.g., can study results be applied to the population).

Each person exported the citations that they considered to be the most relevant into their “article selection” RefWorks folder and Microsoft Excel spreadsheet. The articles included in each person's “article selection” folder were then combined into one RefWorks folder and de-duplicated.

The critical appraisal expert reviewed the combined RefWorks folder and determined—based on study design, direct applicability, and actionability—which articles to include in the Evidence Brief. The articles selected by each individual as most relevant were assessed to determine the proportion included in the Evidence Brief. As the critical appraisal expert selected articles for inclusion in the Evidence Brief, she documented the reasons why certain articles were not included. Examples of reasons for exclusion were protocol only, not primary literature, not head-to-head comparison, included in systematic review, and inappropriate study design.

References included in each Evidence Brief were analyzed for inclusion on the spreadsheets containing all search results and selected article citations for each individual. Numbers of citations that were captured in literature searches and of articles selected by each individual were recorded in a spreadsheet where percentages were generated for comparison.

## RESULTS

Analysis of each EBP team member's performance of literature searching and article selection was completed for each Evidence Brief. For each individual, the percentage of references captured per search strategy and percentage of articles selected for inclusion in each Evidence Brief were calculated.

### Literature searching skills analysis

**Figure 2:**
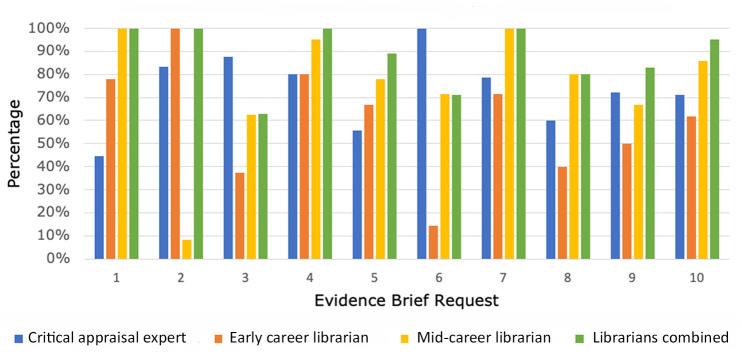
Percentage of references capture through literature search

Across all Evidence Brief requests, the mid-career librarian performed the most comprehensive literature searches and captured the highest number of articles that ultimately ended up being included in the Evidence Briefs (75%), followed by the critical appraisal expert (73%) and then the early career librarian (60%). Analysis of each individual's performance on the literature searching component for each Evidence Brief request can be found in Figure 2.

Results of both librarians' literature searches were combined and compared to those of the critical appraisal expert to examine results of librarian-combined to nonlibrarian-developed literature searches. When combined, the search strategies of both librarians retrieved an average of 88% of articles that were selected to be included in the Evidence Briefs, compared to 73% retrieved by the critical appraisal expert. Results of librarian-combined to nonlibrarian-developed search strategies can be found in Figure 2.

### Article selection skills analysis

**Figure 3:**
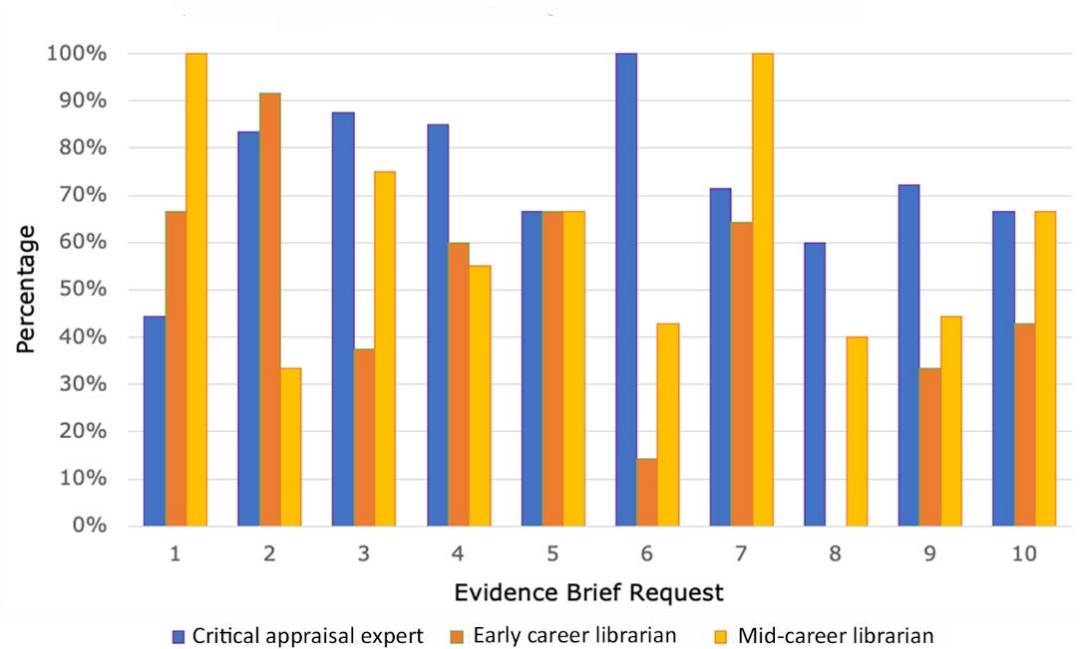
Percentage of references included in Evidence Briefs

Across all Evidence Brief requests, the critical appraisal expert performed best on the article selection portion of the project and identified the highest number of relevant articles that were included in Evidence Briefs (74%), followed by the mid-career librarian (62%) and then the early career librarian (48%). Analysis of each individual's performance on the article selection component for each Evidence Brief request can be found in Figure 3.

## DISCUSSION

This project provided a formalized method of assessing the literature searching and article selection skills of each member of the Value Institute's EBP team. This is particularly important because the quality of Evidence Briefs that are provided to clinicians solely depends on the evidence that is located and selected for inclusion. While this project focused on Evidence Briefs, librarians are routinely involved in developing hospital guidelines, systematic reviews, and other projects where quality is highly dependent upon expert searching and article selection skills.

This study appears to be the first published evaluation of literature searching and article selection skills of an EBP team. Although previous studies examined the impact of including librarians on clinical teams [7, 8], those studies compared clinical teams that included librarians to those with no librarians involved and did not directly compare skills between librarians and clinicians. Additionally, one report compared the article selection skills of librarians and clinicians [9], but the search results were focused on educational research topics and were not clinical in nature.

This project illustrated the skill levels of each individual and led to improvements in the Evidence Brief request work flow. The new work flow allows each EPB team member to focus on their individual strengths, improves quality, and increases productivity through the time saved. A comparison of the work flow before and after the QI project can be found in supplemental Appendix B.

The results indicate that the combined librarians' searches are equally or more effective than the searches of any individual. Going forward, the librarians have worked together to develop search strategies as opposed to one individual completing search strategies. This ensures that a comprehensive search strategy is developed and provides an opportunity for mentorship. After the QI project, the early career librarian develops the initial search, which is then tested and modified as appropriate by the mid-career librarian. Only modifying literature searches, instead of having to start from the beginning, saves time for the mid-career librarian. The early career librarian spends more time conducting the literature searches, as she develops the initial strategy for each request, and this increased exposure and practice allows her to continue developing her clinical searching skills.

The librarians have implemented a formal mentoring program, where after each search is finalized, both librarians meet and discuss the search and any modifications that have been made. Both librarians are learning from each other through this experience. For example, the mid-career librarian is more familiar with terminology used in the literature to express certain concepts, and the early career librarian has been able to add those terms based on feedback. The early career librarian is also the liaison to the affiliated College of Pharmacy and is more knowledgeable when searching for requests that involve medications. Thus, she has been able to share knowledge specific to searching for drug information with the mid-career librarian. Since the results of the project show the librarian-developed search strategies were most effective, the critical appraisal expert no longer runs preliminary searches, saving her time as well.

The results demonstrate that the critical appraisal expert is best at article selection. Therefore, the librarians are no longer spending time identifying articles that they believe are relevant for inclusion in Evidence Briefs. The critical appraisal expert began documenting exclusion criteria during the article selection phase, which resulted in more transparency for the team and the requestors. Thus, the documentation of exclusion criteria is now consistent practice.

Additionally, new project management methods were introduced and ultimately adopted for Evidence Brief requests. Prior to this project, requests were manually tracked by the critical appraisal expert, and all communication was conducted via email. The librarians, who previously utilized the Springshare LibAnswers platform to answer patron requests via tickets and to record reference statistics, suggested using the platform for Evidence Brief requests as a project management tool. Doing so allows requests to be tracked in a central location, and ownership can be assigned and transferred seamlessly, increasing the clarity of team members' responsibilities during each phase of the process. This system also has internal notes and statistics-tracking features that enhance project management of the requests. Statistics on this service are more accurately tracked since all communication is being conducted in LibAnswers, which is valuable for library administration and used for organization and business planning.

One potential limitation of this project was that there was only one critical appraisal expert on the EBP team; therefore, the team was unable to enlist an impartial critical appraisal expert to evaluate each individual's article selection skills. The EBP team attempted to mitigate potential bias by initiating a time delay between the critical appraisal expert's individual selection of articles and selection from the combined results, as well as documenting exclusion criteria.

There are multiple issues that limit the generalizability of this QI project and Evidence Brief service. The critical appraisal expert has above average search skills for a nonlibrarian. The mid-career librarian and critical appraisal expert have taught EBP courses together for years, honing the literature searching skills of the critical appraisal expert. Also, the Value Institute is a unique department with a preexisting link between the quality department and medical library of an academic medical center. This limits the ability of another organization without this type of cross-collaboration to immediately apply the same process to create or improve an evidence synthesis service.

While not many libraries have formally established EBP teams such as this one, other libraries are able to implement this process to assess the quality of librarians' literature searching and article selection skills. The EBP team feels this is a particularly effective method of establishing the literature searching skills of early career librarians, compared to those who are more experienced, and development of personalized training programs can be established based upon the results. This QI project required little additional work from the librarians outside of standard work processes, and the EBP team gained valuable insight that led to improvements in previously established work processes.

## Data Availability

Data from this project can be found in the Open Science Framework at https://osf.io/davmp/?view_only=df3ae10fce944c488d80d7bdbe0b9f6f.

## SUPPLEMENTAL FILES

**Appendix A:** Example Evidence Brief

**Appendix B:** Evidence Brief request work flow before and after quality improvement project

**Appendix C:** Search strategy comparison
