# Supplementary material for: Evaluation of literature searching and article selection skills of an evidence-based practice team
Source: J Med Libr Assoc. 2020 Jul 1;108(3):487–93. doi: 10.5195/jmla.2020.865 (PMC7441910; doi:10.5195/jmla.2020.865)
Supplement: Supplementary file 1 — Appendix A: Example Evidence Brief [file jmla-108-3-487-s01.pdf]

JMLA Journal of the Medical Library Association

SC
Supplemental content to
J Med Libr Assoc. Jul;108(3):dx.doi.org/10.5195/jmla.2020.865
www.jmla.mlanet.org
© Jones, Brennan, Davis 2020

Evaluation of literature searching and article selection skills of an evidence-based practice team

Emily Paige Jones, AHIP; Emily A. Brennan; Amanda Davis

APPENDIX A
Example evidence brief

Effectiveness of neuromuscular electrical stimulation (NMES) in adult intensive care unit (ICU) patients

Ask the question

Question: In adult ICU patients, what is the effect of NMES on functional outcomes and length of stay?

Search for evidence

Databases: PubMed, Scopus

PubMed search strategy: ("Electric Stimulation/methods"[Mesh] OR "Electric Stimulation Therapy/methods"[Mesh] OR "electrical stimulation" OR NEMS) AND ("Intensive Care Units"[Mesh] OR "critical care" OR "intensive care unit" OR ICU) AND ("Polyneuropathies"[Mesh] OR polyneuropathy OR polyneuropathies OR myopathy OR neuropathy)

Filters: Humans, English, Published last 10 years

All 5 systematic reviews found that NMES preserves muscle strength and/or prevents skeletal muscle weakness in critically ill patients. Burke et al. (2016) included a meta-analysis of 3 studies that found that NMES preserved muscle strength (standardized mean difference 0.93, 95% CI 0.51–1.35, p=0.0002), and a meta-analysis of 2 studies by Wageck et al. (2014) found that NMES on quadriceps femoris showed significant effects on muscle strength (standardized mean difference 0.77, 95% CI 0.13–1.40, p=0.02).

Four randomized controlled trials (RCTs) (Akar et al., 2017; Dos Santos et al., 2018; Fossat et al., 2018; Patsaki et al., 2017) published after 2016 evaluated the effect of NMES on both muscle preservation and secondary outcomes like mobility or function, mechanical ventilation, and length of stay. Patsaki et al. (2017) evaluated the effect of NMES plus individualized rehab on hospital length of stay in 128 adult patients within 48 hours of ICU discharge, compared with sham NMES and usual care rehab. While the interventions did not take place in the ICU, they found that **functional status via functional independence measure (90±29 vs. 99±24, p=0.069) and hospital length of stay (22±22 vs. 19±15 days, p=0.35) *did not* differ at hospital discharge between the 2 groups.** Fossat et al. (2018) found that **days of mechanical ventilation, number of mechanical ventilation–free days at 28 days after discharge from ICU, frequency of reintubation within 48 hours, and frequency of delirium was *not significantly different* between a group of patients receiving usual care and another receiving usual care plus daily NMES and in-bed cycling**. There were also no significant differences between the groups when subgrouping for ICU survivors and ICU decedents. Akar et al. (2017) evaluated active extremity mobilization and NMES in ventilated chronic obstructive pulmonary disease (COPD) patients to determine the impacts of these 2 interventions alone and combined on weaning from mechanical ventilation. They noted **no statistically significant difference in ventilator weaning times between the 3**

SC
Supplemental content to
J Med Libr Assoc. Jul;108(3):dx.doi.org/10.5195/jmla.2020.865
www.jmla.mlanet.org
© Jones, Brennan, Davis 2020

groups (active mobilization + NMES: median day 2 vs. NMES: median day 2 vs. active mobilization: median day 4; *p*=0.781). However, there may be clinical importance to a decrease in 2 days when using NMES. There was also a potentially clinically important, but not statistically significant, difference in multiple mobilization parameters in both groups receiving NMES. However, Dos Santos et al. (2018) evaluated the use of NMES plus exercise and NMES alone compared to usual care in 51 mechanically ventilated patients. They found a **significantly shorter duration on mechanical ventilation (*p*=0.007) in the NMES plus exercise group (5.7±1.1 days) and the NMES group (9.0±7.0 days) compared to control (14.8±5.4 days)**. Further research is needed to determine the true effect of NMES on functional, mechanical ventilation and length of stay outcomes.

Five additional RCTs (Dall' Acqua et al., 2017; Kho et al., 2015; Koutsioumpa et al., 2018; Leite et al., 2018; Pandy et al., 2013) have been published that all support the use of NMES for preserving muscle strength. They were not included in this evidence brief because they did not assess for functional outcomes, mechanical ventilation outcomes, or length of stay.

There were nine studies (Akar et al., 2017; Burke et al., 2016; Dos Santos et al., 2018; Fossat et al., 2018; Maffiuletti et al., 2013; Parry et al., 2013; Patsaki et al., 2017; Wageck et al., 2014; Williams et al., 2014) found that addressed the effects of NMES in adult ICU patients. The majority of the studies, including five systematic reviews (Burke et al., 2016; Maffiuletti et al., 2013; Parry et al., 2013; Wageck et al., 2014; Williams et al., 2014), addressed the effects of NMES on muscle strength instead of functional outcomes or length of stay. Only one study (Patsaki et al., 2017) directly addressed the effect of NMES on length of stay.

Supplemental content to
J Med Libr Assoc. Jul;108(3):dx.doi.org/10.5195/jmla.2020.865
www.jmla.mlanet.org
© Jones, Brennan, Davis 2020

Problem, intervention, comparison, outcome (PICO) question: In adult intensive care unit (ICU) patients, what is the effect of neuromuscular electrical stimulation on functional outcomes and length of stay?

Author/date/ journal	*Purpose of study*	*Study design*	*Sample and setting*	*Outcomes*	*Design limitations*
Burke et al., 2016, *Clinical Respiratory Journal*	To review the use of neuromuscular electrical stimulation (NMES) in the critical care setting compared with usual care, under all domains of the World Health Organization International Classification of Functioning, Disability and Health (ICF) framework	Systematic review and meta-analysis	12 studies (11 randomized controlled trials [RCTs], 1 case-control study; 449 patients from the critical care setting)<br>– muscle strength (n=3)<br>Variety of stimulation parameters:<br>– quadriceps femoris (all studies), in addition to peroneus longus, vastus glutei, tibialis anterior, and triceps surae<br>– 1 study targeted brachial biceps<br>– frequencies varied (35 to 100 hertz [Hz]), as did pulse width and intensities of stimulation<br>– studies adjusted until visible	Meta-analysis (n=3) supported **NMES to preserve muscle strength using a fixed-effects model (n=146; standardized mean difference 0.93, 95% CI 0.51–1.35, *p*=0.0002; I²=88%)**<br>NMES effect on body function and structure:<br>– mortality: conflicting effects<br>– ventilation: potentially beneficial effects on duration and weaning period<br>– joint range of motion: potential for improvement	Study limitations=<br>☐ None<br>***Systematic review***<br>☐ Review did not address focused clinical question<br>☐ Search was not detailed or exhaustive<br>☒ Quality of the studies was not appraised or studies were of low quality<br>☒ Methods and/or results were inconsistent across studies

GRADE CRITERIA

Lower quality rating if:

☐ High risk of bias *(When design limitations for one or more criteria impact the quality of studies sufficiently enough to lower confidence in the estimate of effect)*

☒ Studies inconsistent *(When there are differences in the direction of the effect, populations, interventions, or outcomes between studies)*

☒ Studies are indirect

(Your PICO question is quite different from the available evidence in regard to PICO)

☐ Studies are imprecise *(When studies include few patients and few events and thus have wide confidence intervals [CIs] and the results are uncertain)*

☐ Publication bias

(e.g., pharmaceutical company sponsors study on effectiveness of drug)

JMLA Journal of the Medical Library Association

SC
Supplemental content to
J Med Libr Assoc. Jul;108(3):dx.doi.org/10.5195/jmla.2020.865
www.jmla.mlanet.org
© Jones, Brennan, Davis 2020

			muscle contraction or maximum pain toleration<br>– duration ranged from 30 minutes to 60 minutes per day for a variety of time limits (7 days, 30 days, extubation, ICU discharge, voluntary movement)			<u>Increase quality rating if:</u><br>☐ Large effect<br>*(When the relative risk of association between two factors is large or very large)*<br>☐ Dose response<br>*(When the dose-response relationship increases the confidence than an effect is real and substantial)*<br>☐ Plausible confounders<br>*(When plausible residual confounding is directly impacting the magnitude of effect)*<br>Level of evidence for studies as a whole:<br>☐ High<br>☐ Moderate<br>☒ Low<br>☐ Very low
Maffiuletti et al., 2013, *BMC Medicine*	To evaluate the effectiveness of NMES for preventing skeletal-muscle weakness and wasting in critically ill patients, in comparison with usual care	Systematic review	8 RCTs (172 patients)<br>– muscle strength (n=5)<br>– muscle mass (thickness, volume; n=4)<br>Variety of stimulation parameters:<br>– targeted quadriceps, hamstrings, peroneus longus, and glutei<br>– 1 study targeted biceps brachii<br>– frequencies varied (35 to 100 Hz), as did pulse width	**NMES added to usual care proved to be more effective than usual care alone for preventing skeletal muscle weakness in critically ill patients**<br>– **supported by all 5 studies**<br>There is inconclusive evidence for its benefit in prevention of muscle wasting<br>– volume loss and muscle thickness results varied by study	Study limitations=<br>☐ None<br>***Systematic review***<br>☐ Review did not address focused clinical question<br>☐ Search was not detailed or exhaustive<br>☐ Quality of the studies was not appraised or studies were of low quality<br>☒ Methods and/or results were inconsistent across studies	

SC
Supplemental content to
J Med Libr Assoc. Jul;108(3):dx.doi.org/10.5195/jmla.2020.865
www.jmla.mlanet.org
© Jones, Brennan, Davis 2020

			and intensities of stimulation<br>– studies adjusted treatment to individual pain tolerance or set percentage of motor threshold<br>– duration ranged from 7 days to 2 weeks			
Parry et al., 2013, *Critical Care Medicine*	To identify, evaluate, and synthesize the evidence examining the effectiveness and the safety of electrical muscle stimulation in the ICU and the optimal intervention variables	Systematic review	9 studies (8 RCTs, 1 case-control study; 136 patients)<br>– muscle thickness and circumference; muscle strength<br>Variety of stimulation parameters:<br>– targeted quadriceps, peroneus longus, and gastrocnemius<br>– 1 study targeted biceps brachii<br>– frequencies varied (1.75 to 100 Hz), as did pulse width and intensities of stimulation	**Electrical muscle stimulation may be beneficial in preventing muscle wasting in the ICU setting, especially in long-stay patients**<br>– **particularly when administered in long-stay and those with lower acuity**<br>– no benefits were noted in patients with ICU stays < 7 days or in high acuity patients	Study limitations=<br>☐ None<br>***Systematic review***<br>☐ Review did not address focused clinical question<br>☐ Search was not detailed or exhaustive<br>☐ Quality of the studies was not appraised or studies were of low quality<br>☒ Methods and/or results were inconsistent across studies	

Supplemental content to
J Med Libr Assoc. Jul;108(3):dx.doi.org/10.5195/jmla.2020.865
www.jmla.mlanet.org
© Jones, Brennan, Davis 2020

			– studies adjusted treatment to visible contraction or patient's maximum tolerable intensity<br>– duration was variable as was the type of muscle training (interval vs. continuous)			
Wageck et al., 2014, *Medicina Intensiva*	To investigate the applications and effects of NMES in critically ill patients in the ICU	Systematic review and meta-analysis	8 studies (9 articles)<br>– meta-analysis of muscle strength outcome (n=2; 66 patients)<br>Variety of stimulation parameters:<br>– targeted quadriceps, vastus medialis and lateralis, hamstrings, and fibularis longus<br>– 1 study targeted biceps brachii<br>– frequencies varied (1.75 to 100 Hz), as did pulse width and intensities of stimulation	**NMES on quadriceps femoris assessed using Medical Research Council (MRC) scale for muscle strength showed significant effects in favor of NMES (standardized mean difference 0.77, 95% CI 0.13–1.40, *p*=0.02, I^2=56%)**<br>One study reported better performance for mechanical ventilation weaning with NMES, but no difference in length of ICU stay<br>One study reported decreased odds of developing polyneuropathy when using NMES	Study limitations=<br>☐ None<br>*Systematic review*<br>☐ Review did not address focused clinical question<br>☐ Search was not detailed or exhaustive<br>☐ Quality of the studies was not appraised or studies were of low quality<br>☒ Methods and/or results were inconsistent across studies	

JMLA Journal of the Medical Library Association

SC
Supplemental content to
J Med Libr Assoc. Jul;108(3):dx.doi.org/10.5195/jmla.2020.865
www.jmla.mlanet.org
© Jones, Brennan, Davis 2020

			– studies adjusted treatment to visible contraction<br>– duration was variable (4 days, 7 days, until extubation, ICU discharge)			
Williams et al., 2014, *Physiotherapy Theory and Practice*	To evaluate the efficacy of NMES in critically ill patients	Systematic review	8 studies (2 RCTs, 2 randomized interventional trials, 4 prospective observational)<br>– systemic effects, muscle mass, muscle strength<br>Variety of stimulation parameters:<br>– most studies targeted lower limbs, 1 study targeted biceps brachii<br>– frequencies varied (1.75 to 100 Hz), as did pulse width and intensities of stimulation<br>– studies adjusted treatment to visible contraction<br>– duration was variable (4 days, 7 days, until	Systemic effects (n=2):<br>– one study reported reduction in creatinine in urine, and a second reported increases in reperfusion rate, systolic blood pressure (BP) and heart rate after NMES<br>**NMES has potential benefits in improving muscle strength in critically ill patients**<br>– **unable to identify when NMES should be initiated or the most effective protocol**<br>– **current evidence does not identify if NMES has an effect on functional, quality of life, or long-term outcomes**	Study limitations=<br>☐ None<br>***Systematic review***<br>☐ Review did not address focused clinical question<br>☐ Search was not detailed or exhaustive<br>☐ Quality of the studies was not appraised or studies were of low quality<br>☒ Methods and/or results were inconsistent across studies	

JMLA Journal of the Medical Library Association

SC
Supplemental content to
J Med Libr Assoc. Jul;108(3):dx.doi.org/10.5195/jmla.2020.865
www.jmla.mlanet.org
© Jones, Brennan, Davis 2020

			extubation, ICU discharge)	Muscle mass outcomes were varied		
Akar et al., 2017, *Clinical Respiratory Journal*	To investigate the impact of active extremity mobilization and NMES on weaning processes, discharge from hospital, and inflammatory mediators in chronic obstructive pulmonary disease (COPD) patients receiving mechanical ventilation	RCT	30 conscious COPD patients in the ICU for respiratory failure receiving mechanical ventilation<br>– Group 1: active extremity-exercise training + NMES (n=10)<br>– Group 2: NMES only; bilateral upper and lower extremities 5 days/week (n=10)<br>– Group 3: active extremity-exercise only; active or passive range of motion exercises for upper and lower extremities (n=10)<br>– all groups received other components of pulmonary rehab (positioning, postural drainage, bronchial hygiene, deep tracheal aspiration, nutrition, and	NMES + active exercise (*p*=0.014) and NMES alone (*p*=0.046) exhibited statistically significant improvement especially in lower extremity muscle strengths<br>– all 3 groups had significant increases in upper extremity muscle strength following intervention<br>**Ventilator weaning time of the groups was comparable (median: day 2 vs. day 2 vs. day 4; *p*=0.781)**<br>Mobilization parameters were statistically similar between all groups but may be clinically important:<br>Time to sit up assisted in bed (Group 1: 1.25±0.50 days vs. Group 2: 3.33±4.04 days vs. Group 3: 4.40± 3.91 days, *p*=0.712)<br>Time to sit up unassisted in bed (Group 1: 1.50±1.00 days vs. Group 2: 3.66±4.61 days vs.	Study limitations=<br>☐ None<br>***RCT & quasi-experimental studies***<br>☒ Insufficient sample size<br>☐ Lack of randomization<br>☒ Lack of blinding<br>☐ Stopped early for benefit<br>☒ Lack of allocation concealment<br>☐ Selective reporting of measures<br>☐ Large losses to follow up (F/U)	

JMLA Journal of the Medical Library Association

Supplemental content to
J Med Libr Assoc. Jul;108(3):dx.doi.org/10.5195/jmla.2020.865
www.jmla.mlanet.org
© Jones, Brennan, Davis 2020

			psychological support)<br>– monitored from intubation through extubation, death, or discharge home<br>Included patients: COPD patients (stage C or D) for at least 24 hours, without deep vein thrombosis (DVT) or comorbidities<br>Excluded patients: stage D/C from ICU within 48 hours, developed infection, unconscious, hemodynamically unstable<br>Evaluated muscle strength, mobilization duration, and weaning	Group 3: 6.80 +3.96 days, *p*=0.500)<br>Time to sit up assisted at bedside (Group 1: 3.25+2.21 days vs. Group 2: 4.00+5.19 days vs. Group 3: 7.20+4.94 days, *p*=0.402)<br>Time to sit up unassisted at bedside (Group 1: 3.75+2.50 days vs. Group 2: 6.00+4.35 days vs. Group 3: 7.60+4.50 days, *p*=0.304)<br>Time to stand assisted (Group 1: 4.25+2.98 days vs. Group 2: 7.00+4.35 days vs. Group 3:11.0 +5.24 days, *p*=0.671)<br>Time to stand unassisted (Group 1: 5.25+2.62 days vs. Group 2: 8.00+4.35 days vs. Group 3: 12.00 +5.61 days, *p*=0.123)<br>Time to move from bed to chair (Group 1: 5.25+2.62 days vs. Group 2: 8.33+4.04 days vs. Group 3: 12.60+6.30 days, *p*=0.102)			
Dos Santos et al., 2018, *Physiotherapy*	To assess the efficacy of NMES, exercise, and combined therapy (NMES +	RCT	51 patients receiving mechanical ventilation in a single ICU in a	**Duration on mechanical ventilation was significantly shorter (*p*=0.007) in the NMES + exercise group (5.7±1.1**	Study limitations=<br>☐ None		

SC
Supplemental content to
J Med Libr Assoc. Jul;108(3):dx.doi.org/10.5195/jmla.2020.865
www.jmla.mlanet.org
© Jones, Brennan, Davis 2020

Theory and Practice	exercise) on duration of mechanical ventilation in critically ill patients		tertiary hospital in Brazil (2012–2014)<br>– control: 55 minutes 2 times per day; usual care of physical therapy like passive mobilization, positioning and stretching (n=15)<br>– NMES: 55 minutes 2 times per day; simultaneously on rectus femoris, vastus lateralis, and vastus medialis bilaterally (n=11)<br>– exercise: 55 minutes 2 times per day; manual assistance progressing to active and resistance exercises with elastic bands (n=13)<br>– NMES + exercise: 55 minutes 2 times per day; combination of above (n=12)	**days) and NMES group (9.0±7.0 days) in comparison to control (14.8±5.4 days)**<br>– survivors only (n=38): duration of mechanical ventilation was significantly shorter in the NMES + exercise group (5.75±1.3) versus control (12.83±3.6)<br>Duration of sedation was significantly shorter in NMES + exercise (0.6±1.0) and exercise (0.4±0.5) groups compared with controls (5.83±5.1) (survivors only; n=38)	***RCT & quasi-experimental studies***<br>☒ Insufficient sample size<br>☐ Lack of randomization<br>☒ Lack of blinding<br>☐ Stopped early for benefit<br>☐ Lack of allocation concealment<br>☐ Selective reporting of measures<br>☐ Large losses to F/U	

			Period of mechanical ventilation=intubation to day when no need for it in next 48 hours<br><br>Included patients: > 18 years old, mechanical ventilation for < 72 hours, no known neuromuscular disorders<br><br>Excluded patients: cardiopulmonary arrest, end stage cancer, increased intracranial pressure, bone fractures, or skin lesions making NMES difficult, mechanical ventilation > 21 days (n=3 patients)			
Fossat et al., 2018, *Journal of the American Medical Association*	To investigate whether early in-bed leg cycling plus electrical stimulation of the quadriceps muscles added to standardized early rehabilitation would result in greater muscle strength at discharge from the ICU	RCT	312 critically ill adults admitted to a single ICU in France<br><br>– usual care: 10 passive range of motion exercises with each limb joint once every weekday, followed by passive or active exercises and then fully active muscle exercises (n=154)	Median MRC score at ICU discharge did not differ between groups (48 [IQR 29–58] in the intervention group and 51 [IQR 37–58] in the usual care group)<br><br>– median difference −3.0 (95% CI, −7.0 to 2.8, *p*=0.28)<br><br>**Days of mechanical ventilation, # mechanical ventilation-free days at 28 days after discharge from ICU, frequency of**	Study limitations=<br><br>☐ None<br><br>***RCT & quasi-experimental studies***<br><br>☐ Insufficient sample size<br><br>☐ Lack of randomization<br><br>☒ Lack of blinding<br><br>☐ Stopped early for benefit<br><br>☐ Lack of allocation concealment<br><br>☐ Selective reporting of measures	

SC
Supplemental content to
J Med Libr Assoc. Jul;108(3):dx.doi.org/10.5195/jmla.2020.865
www.jmla.mlanet.org
© Jones, Brennan, Davis 2020

			– usual care + 15 minutes in-bed cycling + 50 min electrical stimulation of quadriceps (n=158)<br>– 8% on mechanical ventilation at study inclusion<br>– 18.7% died in the ICU<br>Included patients: > 18 years old, admitted to ICU less than 72 hours before randomization, needed > 48 hours ICU care, independent walking ability, Barthel index > 55 within 15 days prior to ICU admission<br>Excluded patients: pregnant, cardiac arrest, pacemaker or implantable device, acute cerebral disease requiring deep sedation for at least 72 hours, Guillian-Barre syndrome, myasthenia, advanced dementia, venous thromboembolism (VTE) or pulmonary embolism (PE) treated	**reintubation within 48 hours, frequency of delirium, and ICU mortality were not significantly different between the groups**<br>– **there were also no significant differences between the groups when subgrouping for ICU survivors and ICU decedents**<br>Barthel index was not significantly different between the groups after 6 months<br>Health survey scores were not significantly different between the 2 groups for any component (physical functioning, role physical, bodily pain, general health, vitality, social functioning, emotional role, mental health, physical component, mental component)<br>In hospital, 28-day and 6-month mortality rates were not significantly different between the 2 groups	☐ Large losses to F/U	

Supplemental content to
J Med Libr Assoc. Jul;108(3):dx.doi.org/10.5195/jmla.2020.865
www.jmla.mlanet.org
© Jones, Brennan, Davis 2020

			for < 48 hours, contraindication to electrical stimulation, standing or transfer to chair, or low limb amputation<br><br>Global muscle strength assessed using MRC grading system<br><br>– 6 muscle groups on both sides of body) on day of ICU discharge by a blinded physiotherapist<br><br>Per protocol:<br><br>1) received allocated intervention at least 2 days within 3 calendar days following admission<br><br>2) received allocated intervention 80% of weekdays spent in ICU	Neither of the per-protocol analyses showed any significant differences between the 2 groups		
Patsaki et al., 2017, *Journal of Critical Care*	To investigate the effects of NMES along with individualized rehabilitation on muscle strength of ICU survivors	RCT	128 adult patients within 48 hours of ICU discharge at a single hospital in Greece<br><br>– control: daily sham NMES for 55 minutes on lower limbs and usual care (n=65)	ICU-associated weakness (MRC $\leq$ 48) was diagnosed in 36 patients at ICU discharge: NMES group (n=17) and control group (n=19)<br><br>Compliance rates:	Study limitations=<br><br>☐ None<br><br>***RCT & quasi-experimental studies***<br><br>☐ Insufficient sample size<br><br>☐ Lack of randomization<br><br>☐ Lack of blinding	

© Jones, Brennan, Davis 2020

			– intervention: daily NMES for 55 minutes on lower limbs and individualized rehab 5 days/week (n=63)<br>– from ICU discharge until hospital discharge<br>– patients who died during the study, refused to continue the intervention, or were readmitted to the ICU were assigned scores of 0 for strength testing (MRC and hand grip) at hospital discharge<br>Included patients: > 18 years old, were on mechanical ventilation for > 72 hours, level of consciousness adequate to respond to specific orders (open/close eyes, nod head, etc.)<br>Excluded: pregnancy, body mass index (BMI) > 35 kg/m^2, preexisting neuromuscular	– 86% for NMES and sham NMES<br>– 82% for individualized rehab and 74% for usual care rehab<br>**MRC (48±21 vs. 50±18, *p*=0.53), handgrip (14±13 vs. 16±11, *p*=0.46) functional status via functional independence measure (90±29 vs. 99±24, *p*=0.069) and hospital length of stay (22±22 vs. 19±15 days, *p*=0.35) did not differ at hospital discharge between groups**<br>In the patients with ICU-associated weakness (MRC ≤ 48), significant improvement of muscle strength was shown in the NMES group by 2 weeks post-ICU (change in MRC%: 59%±54% vs. 30%±20%, *p*=0.05)	☐ Stopped early for benefit<br>☒ Lack of allocation concealment<br>☐ Selective reporting of measures<br>☐ Large losses to F/U	

SC
Supplemental content to
J Med Libr Assoc. Jul;108(3):dx.doi.org/10.5195/jmla.2020.865
www.jmla.mlanet.org
© Jones, Brennan, Davis 2020

			disease, technical restrictions for NMES, terminal disease, pacemaker, trauma to spine<br>Individualized rehab=passive and active range of motion, strength with resistance when tolerated, functional exercises, ambulation, balance exercises (40 minutes/weekday)<br>Usual care=strength without resistance, functional exercises and ambulation only with minimal assistance (20 minutes/weekday)<br>Muscle strength assessed using MRC grading system<br>– 6 muscle groups on both sides of body)<br>Randomization stratified by age (≤ or > 50 years old) and MRC (≤ or > 48)			

SC
Supplemental content to
J Med Libr Assoc. Jul;108(3):dx.doi.org/10.5195/jmla.2020.865
www.jmla.mlanet.org
© Jones, Brennan, Davis 2020

REFERENCES

Akar O, Günay E, Ulasli SS, Ulasli AM, Kacar E, Sariaydin M, Solak Ö, Celik S, Ünlü M. Efficacy of neuromuscular electrical stimulation in patients with COPD followed in intensive care unit. Clin Respir J. 2017 Nov;11(6):743–50. DOI: http://dx.doi.org/10.1111/crj.12411.

Burke D, Gorman E, Stokes D, Lennon O. An evaluation of neuromuscular electrical stimulation in critical care using the ICF framework: a systematic review and meta-analysis. Clin Respir J. 2016;10(4):407–20. DOI: http://dx.doi.org/10.1111/crj.12234.

Dall' Acqua AM, Sachetti A, Santos LJ, Lemos FA, Bianchi T, Naue WS, Dias AS, Sbruzzi G, Vieira SR; MoVe-ICU Group. Use of neuromuscular electrical stimulation to preserve the thickness of abdominal and chest muscles of critically ill patients: a randomized clinical trial. J Rehabil Med. 2017 Jan 19;49(1):40–8. DOI: http://dx.doi.org/10.2340/16501977-2168.

Dos Santos FV, Cipriano Jr. G, Vieira L, Güntzel Chiappa AM, Cipriano GBF, Vieira P, Zago JG, Castilhos M, da Silva ML, Chiappa GR. Neuromuscular electrical stimulation combined with exercise decreases duration of mechanical ventilation in ICU patients: a randomized controlled trial. Physiother Theory Prac. 2018 Oct 15:1–9. DOI: http://dx.doi.org/10.1080/09593985.2018.1490363.

Fossat G, Baudin F, Courtes L, Bobet S, Dupont A, Bretagnol A, Benzekri-Lefèvre D, Kamel T, Muller G, Bercault N, Barbier F, Runge I, Nay MA, Skarzynski M, Mathonnet A, Boulain T. Effect of in-bed leg cycling and electrical stimulation of the quadriceps on global muscle strength in critically ill adults: a randomized clinical trial. JAMA. 2018 Jul 24;320(4):368–78. DOI: http://dx.doi.org/10.1001/jama.2018.9592.

Kho ME, Truong AD, Zanni JM, Ciesla ND, Brower RG, Palmer JB, Needham DM. Neuromuscular electrical stimulation in mechanically ventilated patients: a randomized, sham-controlled pilot trial with blinded outcome assessment. J Critic Care. 2015 Feb;30(1):32–9. DOI: http://dx.doi.org/10.1016/j.jcrc.2014.09.014.

Koutsioumpa E, Makris D, Theochari A, Bagka D, Stathakis S, Manoulakas E, Sgantzos M, Zakynthinos E. Effect of transcutaneous electrical neuromuscular stimulation on myopathy in intensive care patients. Am J Crit Care. 2018 Nov;27(6):495–503. DOI: http://dx.doi.org/10.4037/ajcc2018311.

Leite MA, Osaku EF, Albert J, de Macedo Costa RL, Garcia AM, do Nascimento Czapiesvski F, Ogasawara SM, Bertolini GRF, Jorge AC, Duarte PAD. Effects of neuromuscular electrical stimulation of the quadriceps and diaphragm in critically ill patients: a pilot study. Crit Care Res Pract. 2018 Jul 8:1–8. DOI: http://dx.doi.org/10.1155/2018/4298583.

Maffiuletti NA, Roig M, Karatzanos E, Nanas S. Neuromuscular electrical stimulation for preventing skeletal-muscle weakness and wasting in critically ill patients: a systematic review. BMC Med. 2013 May 23;11:137. DOI: http://dx.doi.org/10.1186/1741-7015-11-137.

Pandey DP, Babu R, Sharma US. Electrical muscle stimulation (EMS) preserve muscle strength in critically ill patients: a pilot study. Indian J Physiother Occupa Ther. 2013 Jul–Sep;7(3):71–5. DOI: http://dx.doi.org/10.5958/j.0973-5674.7.3.068.

Parry SM, Berney S, Granger CL, Koopman R, El-Ansary D, Denehy L. Electrical muscle stimulation in the intensive care setting: a systematic review. Crit Care Med. 2013 Oct;41(10):2406–18. DOI: http://dx.doi.org/10.1097/CCM.0b013e3182923642.

SC
Supplemental content to
J Med Libr Assoc. Jul;108(3):dx.doi.org/10.5195/jmla.2020.865
www.jmla.mlanet.org
© Jones, Brennan, Davis 2020

Patsaki I, Gerovasili V, Sidiras G, Karatzanos E, Mitsiou G, Papadopoulos E, Christakou A, Routsi C, Kotanidou A, Nanas S. Effect of neuromuscular stimulation and individualized rehabilitation on muscle strength in intensive care unit survivors: a randomized trial. J Crit Care. 2017 Aug;40:76–82. DOI: http://dx.doi.org/10.1016/j.jcrc.2017.03.014.

Wageck B, Nunes GS, Silva FL, Damasceno MC, de Noronha M. Application and effects of neuromuscular electrical stimulation in critically ill patients: systematic review. Med Intensiva. 2014 Oct;38(7):44–54. DOI: http://dx.doi.org/10.1016/j.medin.2013.12.003.

Williams N, Flynn M. A review of the efficacy of neuromuscular electrical stimulation in critically ill patients. Physiother Theory Pract. 2014 Jan;30(1):6–11. DOI: http://dx.doi.org/10.3109/09593985.2013.811567.
