# Supplementary material for: Evaluation of literature searching and article selection skills of an evidence-based practice team
Source: J Med Libr Assoc. 2020 Jul 1;108(3):487–93. doi: 10.5195/jmla.2020.865 (PMC7441910; doi:10.5195/jmla.2020.865)
Supplement: Supplementary file 2 — Appendix B: Evidence Brief request work flow before and after quality improvement project [file jmla-108-3-487-s02.pdf]

Journal of the Medical Library Association

Supplemental content to
J Med Libr Assoc. Jul;108(3):dx.doi.org/10.5195/jmla.2020.865
www.jmla.mlanet.org
© Jones, Brennan, Davis 2020

Evaluation of literature searching and article selection skills of an evidence-based practice team

Emily Paige Jones, AHIP; Emily A. Brennan; Amanda Davis

APPENDIX B

Evidence Brief request work flow before and after quality improvement project

Before quality improvement (QI) project	After QI project
1. Evidence Brief is requested<br>a. Critical appraisal expert reviews request and seeks clarification from the requestor if necessary<br>b. A preliminary literature search is executed to gauge scope of available literature<br>c. Request is emailed to the mid-career librarian	1. Evidence Brief is requested<br>a. Critical appraisal expert reviews request and seeks clarification from the requestor if necessary<br>b. Request is entered into LibAnswers queue and assigned to the early career librarian
2. Librarian performs literature searches in appropriate databases and compiles relevant references in RefWorks	2. Early career librarian performs initial search strategies<br>a. Request is transferred to the mid-career librarian via LibAnswers
3. Librarian emails databases searched and search strategies, and shares RefWorks folder with critical appraisal expert	3. Mid-career librarian reviews searches and tests various modifications, before finalizing<br>a. Request is transferred back to the critical appraisal expert<br>b. Both librarians meet to discuss the search and any modifications
4. Critical appraisal expert reviews results and, if needed, executes librarian's search strategies to identify additional articles	4. Critical appraisal expert executes finalized search<br>a. Relevant articles are identified and appraised
5. Critical appraisal expert evaluates relevant articles using the GRADE criteria	5. Critical appraisal expert creates and shares Evidence Brief with clinical requestor
6. Critical appraisal expert creates and shares Evidence Brief with clinical requestor	
