# Supplementary material for: Evaluation of literature searching and article selection skills of an evidence-based practice team
Source: J Med Libr Assoc. 2020 Jul 1;108(3):487–93. doi: 10.5195/jmla.2020.865 (PMC7441910; doi:10.5195/jmla.2020.865)
Supplement: Supplementary file 3 — Appendix C: Search strategy comparison [file jmla-108-3-487-s03.pdf]

JMLA Journal of the Medical Library Association

SC
Supplemental content to
J Med Libr Assoc. Jul;108(3):dx.doi.org/10.5195/jmla.2020.865
www.jmla.mlanet.org
© Jones, Brennan, Davis 2020

Evaluation of literature searching and article selection skills of an evidence-based practice team

Emily Paige Jones, AHIP; Emily A. Brennan; Amanda Davis

APPENDIX C

Search strategy comparison

Question: In adult intensive care unit (ICU) patients, what is the effect of neuromuscular electrical stimulation on functional outcomes and length of stay?

Critical appriasal expert

PubMed: ("Intensive Care Units"[Mesh] OR ICU OR "critical care") AND ("Respiration, Artificial"[Mesh] OR ventilat*) AND ("Electric Stimulation/methods"[Mesh]) OR "Electric Stimulation Therapy/methods"[Mesh] OR "neuromuscular electrical stimulation")

Filters: English, published in last 10 years

Scopus: (“intensive care unit” OR ICU OR “critical care”) AND (“artificial respiration” OR ventilat*) AND “neuromuscular electrical stimulation”

Filters: English,published 2009-2018, Academic Journals

Total search results: 64

Search captured 56% of articles included in the Evidence Brief

Early career librarian

PubMed: ("Electric Stimulation Therapy"[Mesh] OR "electrical stimulation" OR NEMS) AND ("Intensive Care Units"[Mesh] OR "critical care" OR "intensive care unit" OR ICU) AND ("Polyneuropathies"[Mesh] OR polyneuropathy OR polyneuropathies OR myopathy OR neuropathy)

Filters: English, published in the last 10 years

Scopus: ("electrical stimulation" OR NEMS) AND ("critical care" OR "intensive care unit" OR ICU) AND (polyneuropathy OR polyneuropathies OR myopathy OR neuropathy)

Filters: English, published 2009-2019

Total search results: 40

Search captured 67% of articles included in the Evidence Brief

Mid-career librarian

PubMed: (neuromuscular electrical stimulation OR neuromuscular electrostimulation OR "Electric Stimulation"[Mesh]) AND ("Intensive Care Units"[Mesh] OR "intensive care" OR "critical care” OR “critical illness” OR “critically ill”) AND (critical polyneuropathy OR "Polyneuropathies"[Mesh] OR neuropathy OR myopathy)

FIlters: English

Scopus: (electrical stimulation OR neuromuscular electrostimulation) AND ("intensive care" OR "critical care” OR “critical illness” OR “critically ill”) - AND NOT DBCOLL(medl*)

Filters: English

Total search results: 145

Search captured 78% of articles included in the Evidence Brief
